# Estimation of bargaining effect in the decision of monetary compensation of executive in investment bank: Evidence from China

**DOI:** 10.1371/journal.pone.0283771

**Published:** 2023-03-30

**Authors:** Lina Yu, Hua Zhao

**Affiliations:** 1 School of Economics and Management, Changsha University of Science and Technology, Changsha, Hunan, China; 2 School of Economics and Management, Changsha Normal University, Changsha, Hunan, China; Yunnan Technology and Business University, CHINA

## Abstract

Though numerous empirical and theoretical studies have been conducted on the determinants and effects of executive compensation, empirical evidence regarding the bargaining effect on the monetary compensation decisions of executives, especially in a large emerging economy such as China, remains scarce. In this study, a two-tier stochastic frontier and endogenous correction model was developed to quantitatively estimate the bargaining effect on the monetary compensation decisions of investment bank executives. Our study is the first to provide comprehensive empirical evidence that bargaining between investment banks and executives in China significantly affects the compensation decisions of executives. In the bargaining process, investment banks are more proficient than executives, and the comprehensive bargaining effect tends to lower the negotiated compensation of executives. The bargaining effect exhibited obvious heterogeneity in the characteristics of executives and investment banks. When these characteristics tend to augment the bargaining power of executives, the negotiated compensation exhibits a limited decrease; when these characteristics augment the bargaining power of investment banks, the negotiated compensation decreases substantially. Our results provide deep insight into factors that determine executive compensation and help compensation designers of investment banks better understand and design executive pay packages.

## Introduction

Owing to the relaxation of the regulation of the financial industry in China since the late twentieth century, the monetary compensation (MC) of financial executives has increased considerably and exceeded the executive salary scale in other sectors. The salary figures of investment banks (IBs) in Shanghai and Shenzhen from 2008 to 2020 indicate that the average salary of executives increased from 0.50064 to 1.46977 million CNY, with a cumulative increase of 193.578% in 13 years. In this period, the average MC (1.28378 million CNY) of senior executives in IBs was even higher than those in listed commercial banks (0.97231 million CNY). For example, the average MC of executives in CITIC Securities is 2.6278 million CNY, which is the highest in the IB sector; also, it is higher than that of Ping An Bank (2.11281 million CNY), which has the highest compensation figure among commercial banks. The stringent disclosure regulations set by the securities regulatory department of China have made it mandatory to disclose these sums to the public. However, such disclosures foment a sentiment of injustice. Therefore, numerous proposals have been propagated to limit the compensation of financial enterprise executives.

**Fig 1:**
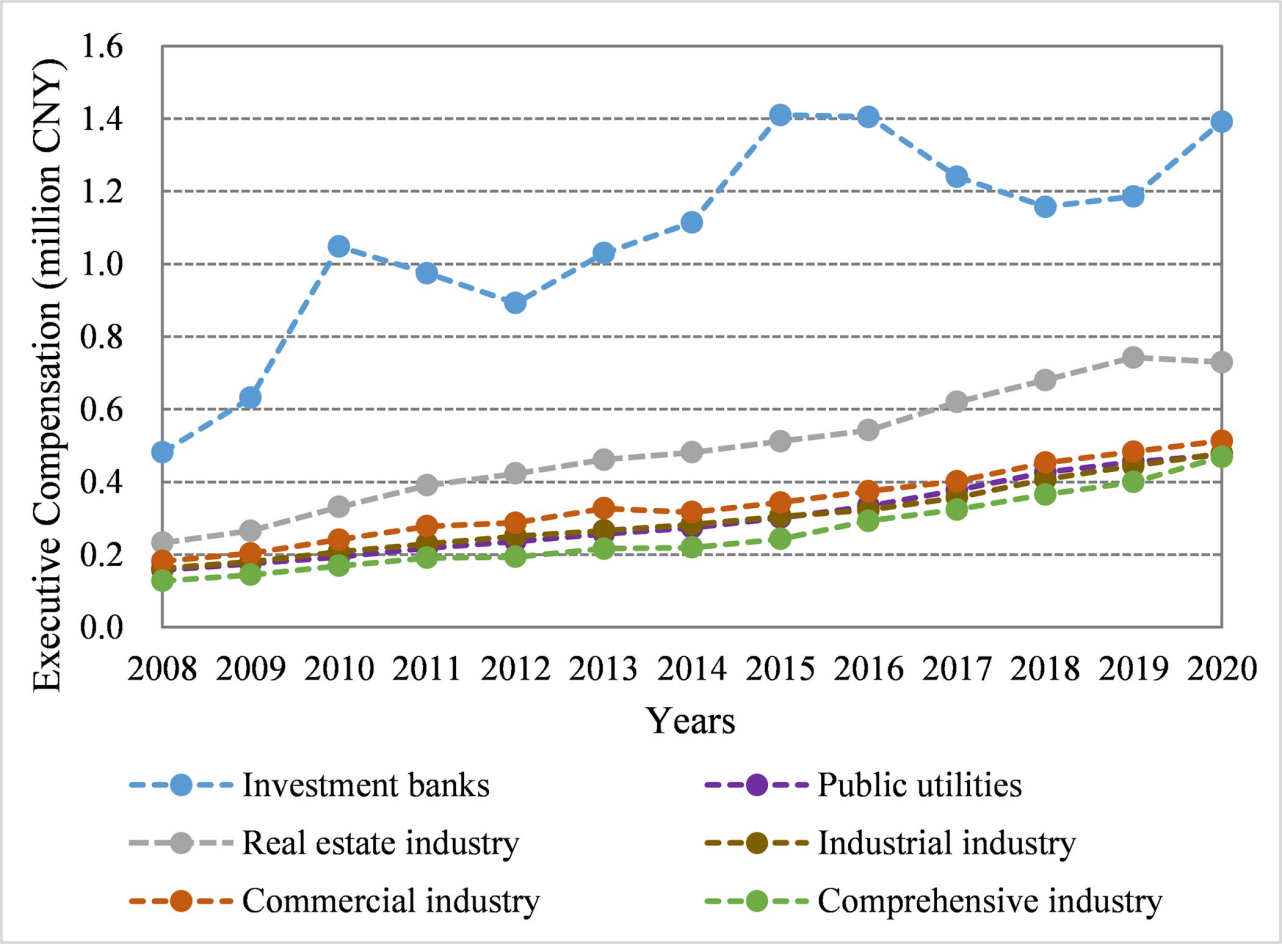
Average monetary compensation of executives in China by industry.

The China Securities Regulatory Commission classifies China’s listed companies into the financial industry, public utilities, real estate industry, industrial industry, commercial industry, and comprehensive industry. Fig 1 presents the changing trend of the average MC of executives in China by industry. It can be seen that executive compensation in all industries has gradually increased from 2008 to 2020, and the compensation figure in IBs is consistently larger than that of other sectors. In China, the IB industry occupies a dominant position in finance. Generally, this pattern of executive compensation is consistent with previous evidence on China’s financial industry [1].

**Fig 2:**
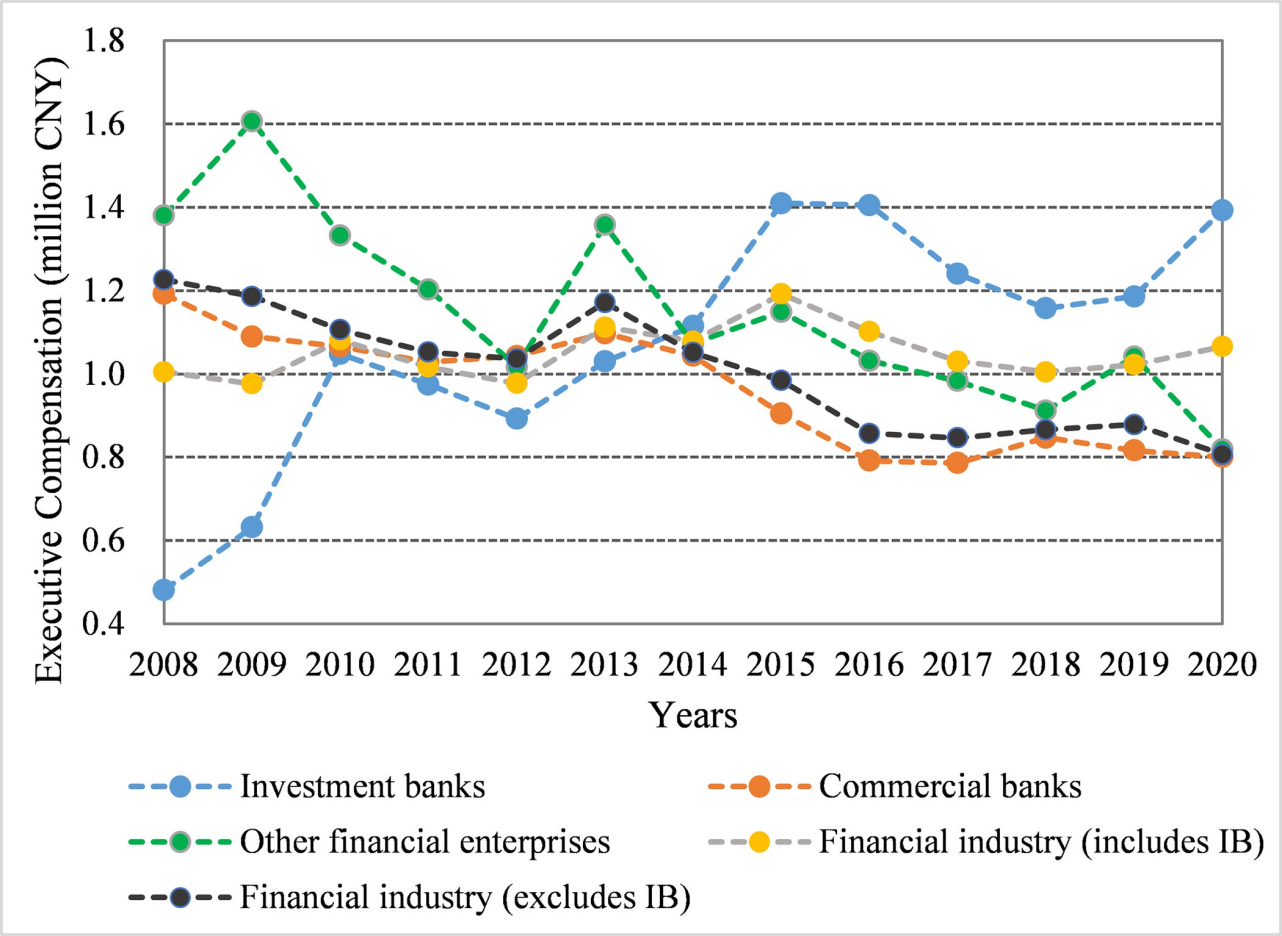
The average monetary compensation of executives in the Chinese financial industry.

The financial industry sample is categorized into IBs, commercial banks, and other financial enterprises. Fig 2 illustrates the changes in the calculated average MC of the financial sub-sectors over years during our full sample period. After the Ministry of Finance of China issued the “Notice on Relevant Issues of Salary Administration for Company’s Executives of State-holding Financial Enterprises” in early 2009, the compensation of executives in state-holding financial enterprises was formally limited to balance the income levels of banks and the social average [2]. With the payment to bank executives limited by the government, the average MC of executives in the financial industry decreased from 2010 to 2012. However, the average executive compensation in the financial industry increased in 2013 and decreased again in 2014. This changing trend is rational because of the implementation of compensation restrictions. The average executive compensation of IBs was approximately 1.115 million CNY in 2014, which fluctuated and increased to approximately 1.392 million CNY in 2020, which exceeded the average executive compensation of the financial industry; the executive compensation of commercial banks and other financial enterprises exhibited a general downward trend during this period. Since 2014, Internet finance developed rapidly in China, and large Internet enterprises such as Alibaba and Tencent have been widely involved in financial businesses such as deposit, credit, and insurance. Internet finance has intensified competition in the financial service market in China, affecting the profitability of conventional financial businesses and threatening the survival of commercial banks and other financial enterprises [3]. The People’s Bank of China issued the “Guidance on Internet Finance Industry” in 2015, which restricted the entry of Internet enterprises into securities businesses. Thus, the development of Internet finance does not marginalize the core business of IBs, and the average compensation of IB executives is less affected.

Unbalanced salary structures and the overpayment of finance executives seriously weaken the internal supervision willingness of IBs. As a result, decision-makers and executives may focus on short-term benefits to maximize their interests, causing the MC incentive policy for executives to fail to maximize the firm value [4]. Academia and industry analyst believe that the unreasonable salary system of financial enterprises is the cause of systemic financial crises [5]. After the global financial crisis of 2008, the financial industry focused on establishing remuneration and performance appraisal committees to review compensation contract designs and performance appraisals, thus ensuring compensation incentives and firm values. After the Political Bureau of the Central Committee of the Communist Party of China issued the “Reform Plan for the Remuneration System of the Principals of Centrally Governed Enterprises” in 2015, the policy of limiting the salaries of executives in the financial industry achieved excellent results. The average growth rate of executive MC during 2008–2014 was as high as 66.252%, which subsequently decreased to 4.553% during 2015–2020. However, financial analysts have suggested that the compensation contract, the most crucial aspect of corporate governance, can alleviate internal principal-agent conflicts and increase the enterprise value, whereas interventions in the executive compensation contracts of financial institutions could reduce the enterprise value [6,7].

The central governance for reforming the payment structure of Chinese financial institutions aims to improve the internal and external governance of financial institutions, design an effective compensation contract mechanism, alleviate the internal principal-agent conflict, decrease the risk level, and increase the enterprise value. Abundant studies have revealed that a perfect compensation contract design needs to systematically grasp decisive factors, including the executive’s job characteristics, capability characteristics, enterprise characteristics, regional differences, and time effects [8–11]. Few studies have emphasized the relationship between bargaining over the compensation setting process and executive compensation. For example, Hermalin and Weisbach (1998) pointed out that the chief executive officer (CEO) with superior bargaining power can considerably influence the board of directors in various decisions including their compensation [12]. Essen et al. (2012) found that when CEOs have bargaining power over the compensation-setting process, they receive significantly higher levels of compensation [13]. Shin (2014) provided empirical evidence that the bargaining power of labor affects executive compensation in US enterprises, and the effect is mainly achieved through the union’s negotiations on workers’ payment [14]. Bova and Yang (2017) developed a model to demonstrate that the bargaining power and equity-based compensation of employees are related [15]. However, these studies are limited to theoretical analysis or existence tests, and relatively little is known about how and to what extent bargaining affects executive compensation. Meanwhile, systematic analysis concerning the effect of bargaining on executive compensation contract design remains scarce. Moreover, the institutional background and labor market characteristics of China, a large emerging economy, vary from those of capitalist countries. Thus, the bargaining effect on executive compensation in China needs to be investigated empirically.

In this research, the establishment of the sample focused on Chines IBs because the average compensation of the executives in the IB industry is considerably higher than that of other Chinese industries (see Figs 1 and 2), indicating the potential bigger bargaining space in the compensation setting process of IB executives. Using a sample of Chinese-listed IBs from 2008 to 2020, this paper fills a gap in the literature by estimating the bargaining effect on the decision of executive MC in Chinese IB. First, a comprehensive theoretical analysis was conducted to illustrate the bargaining between executives and IBs in the compensation setting process, and the bargaining impact mechanism was established. Second, such bargaining effect was quantitatively evaluated by constructing a novel two-tier stochastic frontier and endogenous correction model. When constructing this model, a major concern in the study on determinants of executive compensation like ours is the difficulty in measuring equity-based compensation. Therefore, this paper excluded the sample of equity-holding executives, which accounts for 4.699% of the whole sample to alleviate the endogeneity problem caused by indicator-measurement bias. Moreover, since 4.699% of samples were removed, considering the potential endogeneity problem caused by such sample selection bias, the Heckman two-stage correction method was applied to modify the two-tier stochastic frontier model following the guidelines by Heckman (1979). Finally, based on the executive’s capability characteristics, job characteristics, and enterprise characteristics, a comprehensive heterogeneity analysis of the bargaining effects was conducted on different bargainer groups. Our empirical results indicated that the bargaining between IBs and executives considerably affects the MC decisions of executives. On average, IBs are more proficient at bargaining than executives, and the comprehensive bargaining effect reduces the executive compensation in Chinse IB. Furthermore, it was determined that the degree of the effect of bargaining on executive compensation varied with the characteristics of executives and IBs. When these characteristics tend to augment the bargaining power of executives, the negotiated MC exhibits a limited decrease; when these characteristics augment the bargaining power of IBs, the negotiated MC decreases significantly.

This study contributes to the growing literature on the determinants and impacts of executive compensation. First, previous literature only documented determinants of executive compensation, including the executive’s capability, job, and enterprise characteristics. Few studies have investigated the labor’s bargaining power over the compensation setting process. To the best of our knowledge, this study is the first to provide comprehensive empirical evidence of the bargaining effect on the executive compensation decision in Chinese IBs. Bargaining is proved to be critical for executive compensation, and the effect tends to lower the compensation of IB executives. Second, although the bargaining power is affected by the specific characteristics of the participant in bargaining, the heterogeneity of bargaining effects over the compensation setting process on various bargainer groups has not been extensively examined. Our research adopted a heterogeneity analysis to estimate and compare the bargaining effect on the various types of bargainer groups, thus providing more comprehensive empirical evidence of the bargaining effect on executive compensation than the current literature. Finally, a novel two-tier stochastic frontier and endogenous correction model was developed to quantitatively estimate the bargaining effect on executive compensation. The model solves the potential endogeneity problem caused by the indicator-measurement bias and sample selection bias, which can be used as an effective tool for investigating the bargaining effect on compensation setting.

The rest of this paper is organized as follows. Section 2 presents the literature review. Section 3 details the theoretical analysis of the bargaining effect on executive compensation decisions. Section 4 introduces the empirical methodology, variables selection, and sample selection. Section 5 analyzes the empirical results, and Section 6 performs heterogeneity analysis. Finally, Section 7 presents conclusions and implications.

## Literature review

### Job characteristics and compensation decisions

Managerial power theories state that shareholders cannot fully control the compensation contract design and performance appraisal of executives. Thus, powerful executives receive higher compensation because they can exert greater influence on the judgments of the human resource department and remuneration committee [16]. Studies have demonstrated similar effects, i.e., the maximum self-interest of executives is attained by affecting MC contract decisions [17,18]. Although the executive compensation of financial enterprises is subject to stringent supervision by the Chinese government, and the internal remuneration governance mechanism of financial enterprises is continually improved, powerful executives can still maximize their remuneration by covertly affecting their MC contracts design [19,20]. According to the optimal contracting theory, compensation incentives are exploited to resolve moral hazards, alleviate internal agency conflicts, and motivate executives to work hard for the continuous enhancement of corporate performance [8]. Therefore, the importance and complexity of management work considerably influence MC decisions. Thus, when larger capacity and energy are demanded from executives performing complex and critical tasks, the MC contract design should match job characteristics, and the executive compensation should be higher [21]. These theories indicate that when the job characteristics tend to augment the importance and complexity of executives’ work or increase their power and influence on MC decisions, they are likely to receive higher MC.

### Capability characteristics and compensation decisions

Optimal contracting theories indicate that managerial ability is a critical factor in the design of compensation contracts. In corporate governance practices, compensation incentives are proposed to stabilize the top management team and encourage executives to maximize the enterprise value. Thus, executives with greater managerial ability are likely to receive higher total compensation [9]. This is consistent with the observation that the managerial abilities of executives positively affect MC decisions in the Chinese financial industry [20] and executives with strong management capabilities are likely to have higher compensation [22]. Since executive managerial ability can not be directly measured in practice, researchers typically use personal characteristics to represent working ability. These characteristics include reputation [9], work experience [23], educational experience [24], and social networking ability [25]. Perryman et al. (2016) pointed out that the gender of the executive considerably affects certain abilities [26]. When these characteristics tend to strengthen the working competence of executives, their compensation levels are significantly higher.

### Enterprise characteristics and compensation decisions

Growth-inducing salary rewards are designed to alleviate agent conflicts within the enterprise, decrease the moral hazard of executives, and incentivize executives to do their best work, continuously improving corporate financial performance and maximizing shareholder wealth [27], and the enterprises with better financial performance are more likely to have greater corporate social responsibility performance, thus allowing the enterprise to flourish financially and socially [28]. Studies have shown that MC incentives considerably affect pay-performance sensitivity [10,29]. A test on the sample data of commercial banks in China established a positive correlation between compensation incentives and commercial bank performance [30]. Moreover, because large-scale enterprises are difficult to manage and require more capability and effort, executives in these enterprises generally receive considerably higher compensation [21]. Gabaix et al. (2014) found that executives are given greater compensation in larger firms because these firms require greater managerial ability [31]. Studies have revealed that a high level of corporate governance can reduce agent conflicts within the corporate ladder and incentivize executives to maximize shareholder value [32]. However, the high level of governance increases the dismissal risk of executives [33]. To avoid the negative effects on the motivation of executives by dismissal risks, additional risk premiums should be paid [34].

### Bargaining power and compensation decisions

In organizations, worker wages are determined through the consultation mechanism. In this mechanism, each worker stakes claims to corporate profits based on their contributions to the organizational outcome, and the organization determines worker wages by evaluating their contributions [35]. However, such legitimate claims and organizational decisions to allocate worker wages are inherently contradictory, and the outcome of such discussion depends on the bargaining power of both sides [36]. Similarly, for IB executives, their compensation level is not only affected by executives’ job characteristics, capability characteristics, and enterprise characteristics but also by bargaining between executives and the compensation committee of boards of directors; meanwhile, the attribute and degree of the influence of bargaining on the executive compensation decision depends on the bargaining power of both sides.

The bargaining effect on executive compensation has attracted considerable attention [12–15]. In listed US enterprises, compensation committees determine executive compensation. Although the members of the compensation committee are elected by shareholders and are expected to represent shareholder interests, critics have argued that executives have significantly strong bargaining power in the compensation decision process [14]. Thus, executive compensation exhibits an obvious upward trend despite companies going through scandals and governance reform [37]. However, such evidence should be interpreted with caution given the characteristics of Chinese IBs. In China, since the government is the majority shareholder of IBs, the IB compensation committee is highly regulated by representatives appointed by the government to ensure the implementation of government policies on bank executive compensation [2]. Therefore, aggressive tactics may be adopted by compensation committees for bargaining in the compensation-setting process, which may cause an excessive decrease in executive compensation. Moreover, Chinese IBs with the government as the controlling shareholder are classified as centrally governed enterprises, in which the management is implemented with reference to that of government agencies and characterized by a highly rigid and transparent compensation design system [38], thus reducing the bargaining power of executives to negotiate over wages. In this institutional context, the outcome of bargaining on executive compensation in Chinese IBs may differ from that of US enterprises. In this paper, empirical evidence is provided by estimating the bargaining effect on executive compensation in Chinese IBs.

Extensive studies have been conducted on the determinants and impacts of executive compensation, some of which emphasized the relation between bargaining in the compensation setting process and executive compensation, but empirical evidence regarding the bargaining effect in the compensation setting process remains scarce. Meanwhile, although the effect of bargaining on executive compensation varies with the institutional context of countries, the heterogeneity of bargaining effects in the compensation setting process has received limited attention in the literature, especially in Chinese enterprises. By using a sample of Chinese-listed IBs, this paper aims to contribute to the literature on determinants and impacts of executive compensation by investigating the bargaining effect on executive compensation decisions.

### Theoretical analysis

In the case of information asymmetry, the MC decision of IB executives is considerably affected by job characteristics, capabilities characteristics, enterprise characteristics, and the bargaining behavior between executives and IBs. In the bargaining, executives could increase the MC to maximize their self-interest. By contrast, on the premise of ensuring effective incentives, IBs could decrease MC to save labor costs. The features and degree of the bargaining effect between executives and IBs depend on the bargaining power of both sides. If the bargaining power of executives is stronger than that of IBs, the overall bargaining effect will increase the negotiated MC of executives; otherwise, the overall bargaining effect will reduce the negotiated MC of executives. The negotiated MC is denoted as *S* as follows:

$$S=\underline{S}+\gamma (\bar{S}-\underline{S})$$ (1)

In the above formula, *S* denotes the low end of the expected MC reserved by executives in the bargaining. When the negotiated MC is less than *S*, executives will resign and turn to the job market. $\bar{S}$ denotes the high end of the expected MC reserved by IB in the bargaining. When the negotiated MC is higher than $\bar{S}$, IB will stop the recruitment process and search for new candidates. Here, *S* and $\bar{S}$ are the private information of executives and IB, respectively. Moreover, *γ* represents the bargaining power of executives. And the strength of the bargaining power is assumed to be proportional to the amount of private information held. The degree to which private information determines MC is defined as 1. When private information held by executives is γ, private information held by IBs is (1 –*γ*). *γ* and (1 –*γ*) are exploited to measure bargaining power. Here, $\bar{S}$–*S* denotes the scale of the overall surplus that can be occupied in the bargaining; *γ*($\bar{S}$–*S*) denotes the surplus occupied by executives with their bargaining power (i.e., the bargaining effect of executives’ bargaining behaviors on MC decisions). Then, the MC unaffected by the bargaining is defined as the benchmark MC (*π*). The benchmark MC cannot be directly measured in practice but can be characterized by feature vectors (*θ*), such as executive’s job characteristics, capability characteristics, and IB’s characteristics. Thus, the benchmark MC can be expressed as *u*(*θ*) = *E*(*π*|*θ*), and the constant condition $\bar{S}$≤*u*(*θ*)≤*S* is satisfied. Therefore, the overall surplus that can be occupied in the bargaining can be categorized into *u*(*θ*)–*S* and $\bar{S}$–*u*(*θ*), where *u*(*θ*)–*S* denotes the expected reservation by executives, and $\bar{S}$–*u*(*θ*) denotes the expected reservation by IBs. The theoretical model in (1) can be represented as:

$$S=u(\theta )+[\underline{S}-u(\theta )]+\gamma [\bar{S}-u(\theta )]-\gamma [\underline{S}-u(\theta )]$$ (2)

By shifting and merging theoretical model (2), the following equation can be obtained:

$$S=u(\theta )+\gamma [\bar{S}-u(\theta )]-(1-\gamma )[u(\theta )-\underline{S}]$$ (3)

In theoretical model (3), the negotiated MC is composed of three parts: *u*(*θ*), the benchmark MC which is characterized by executive’s job characteristics, capability characteristics, and IB’s characteristics. *γ*[*—S –u*(*θ*)] denotes the surplus that executives could occupy, and it represents the extent of increased MC by executives’ bargaining behaviors. Besides, (1 –*γ*)[*u*(*θ*)–*S*] denotes the surplus that IBs could occupy, and it indicates the extent of dropped MC by the bargaining behaviors of IBs. The difference between the occupied surplus by the IB and executives (i.e. the comprehensive bargaining effect) determines whether the MC is increased or decreased. Then, the difference is defined as the net surplus (*NTS*), which is expressed as follows:

$$NTS=(1-\gamma )[u(\theta )-\underline{S}]-\gamma [\bar{S}-u(\theta )]$$ (4)

In theoretical model (4), when the net surplus is greater than 0, the bargaining power of IBs is stronger than that of the executives; thus, the IB obtains the net surplus and decreases the negotiated MC. In this case, the *NTS* denotes the MC reduction amount. When the net surplus is smaller than 0, the bargaining power of executives is stronger than that of the IB; thus, the executives obtain a net surplus and increase the MC. In this case, *NTS* denotes the increased amount of MC.

## Research design

### Two-tier stochastic frontier and endogenous correction model

The bargaining effect on the MC decision exhibits bilateral characteristics. That is, the bargaining effect of executives increases the negotiated MC, whereas the bargaining effect of IBs decreases the negotiated MC. Based on the two-tier stochastic frontier analysis proposed by Kumbhakar and Parmeter (2009) [39], theoretical model (3) can be transformed into the following expression:

$$S_{it}=u(\theta_{it})+\varepsilon_{it}\;\varepsilon_{it}=v_{it}+w_{it}-u_{it}$$ (5)

Theoretical model (5) is a typical two-tier stochastic frontier model, where *S*_*it*_ denotes the negotiated MC; *u*(*θ*_*it*_) denotes the benchmark MC; *i* represents the individual cross-section sample; *t* denotes the sample time series. Here, *ε*_*it*_ is the compound interference term, which consists of three parts: *ν*_*it*_, *w*_*it*_, and *u*_*it*_. Specifically, *ν*_*it*_ represents the common stochastic interference term and obeys a normal distribution; *w*_*it*_ represents the surplus occupied by executives, which satisfies *w*_*it*_ ≥0 and equals *γ*[*—S –u*(*θ*)] in the theoretical model (3); *w*_*it*_ denotes the elevation of the negotiated MC by the bargaining effect of executives. Furthermore, *u*_*it*_ denotes the surplus occupied by IBs and equals (1 –*γ*)[*u*(*θ*)–*S*] in theoretical model (3), which indicates a decrease in the negotiated MC because of the bargaining effect of IBs.

In previous studies on the decisive factors of the MC of executives, precisely measuring the return on equity for executives (i.e. the equity-based compensation for executives) was a concern. To avoid the endogenous problem caused by indicator-measurement bias, this study excluded the shareholding sample of executives, which accounts for 4.699% of the whole sample. Moreover, since 4.699% of samples were removed, considering the potential endogeneity problem caused by such sample selection bias, the Heckman two-stage method was adopted to revise the two-tier stochastic frontier model following the guidelines by Heckman (1979) [40]. Specifically, in the first stage, a binary selection model was constructed to systematically analyze the influencing factors of whether the executives’ shareholding samples are included. The mathematical expression of the binary selection model is as follows:

$$Prob_{it}=k_{it}^{'}\beta +\zeta_{it}$$ (6)

In binary selection model (6), when *Prob*_*it*_ = 1, the shareholding samples of executives are included; when the value of *Prob*_*it*_ is 0, the shareholding samples of executives are excluded. Here, *k*_*it*_ denotes the factors affecting whether the shareholding samples of executives are included. In this study, *k*_*it*_ was selected based on the research work performed by Heckman (1979) [40]. Furthermore, *β* denotes the vector of parameters to be estimated, and *ζ*_*it*_ denotes a stochastic interference term. Based on the regression results of the binary selection model, the inverse mills ratio (*IMR*) can be calculated as follows:

$$IMR_{it}=\phi (k_{it}^{'}\beta )/\varphi (k_{it}^{'}\beta )$$ (7)

In the second stage, the *IMR* was compensated into the two-tier stochastic frontier model to overcome the potential endogeneity problem caused by sample selection bias. In this study, the revised model was called the two-tier stochastic frontier and endogenous correction model, which is expressed as follows:

$$S_{it}=u(\theta_{it})+\tau IMR_{it}+\varepsilon_{it}\;\varepsilon_{it}=v_{it}+w_{it}-u_{it}$$ (8)

Where *IMR*_*it*_ represents the inverse mills ratio, and *τ* denotes a parameter to be estimated. Since the two-tier stochastic frontier and endogenous correction model exhibits a nonlinear structure, the maximum likelihood method was employed to estimate the parameter and the occupied surplus. Considering the convenience of regression estimation and the unilateral distribution characteristics of *w*_*it*_ and *u*_*it*_, *w*_*it*_ and *u*_*it*_ were assumed to obey exponential distribution, i.e., *w*_*it*_ *~* exp(*σ*_*w*_, *σ*^*2*^_*w*_) and *u*_*it*_ *~* exp(*σ*_*w*_, *σ*^*2*^_*w*_). Also, *w*_*it*_ and *u*_*it*_ can be assumed to obey a truncated half-normal distribution or a gamma distribution, but Kumbhakar and Parmeter (2009) [39] pointed out that these setting forms do not considerably affect estimation results. Based on these assumptions, the probability density functions of the compound interference term can be expressed as:

$$f(\varepsilon_{it})=\frac{\exp(a_{it})}{\sigma_{u}+\sigma_{w}}\phi (c_{it})+\frac{\exp(b_{it})}{\sigma_{u}+\sigma_{w}}\int_{-h_{it}}^{\infty }\varphi (z)dz=\frac{\exp(a_{it})}{\sigma_{u}+\sigma_{w}}\phi (c_{it})+\frac{\exp(b_{it})}{\sigma_{u}+\sigma_{w}}\varphi (h_{it})$$ (9)

Based on theoretical model (9), the conditional expectation of the occupied surplus by executives and IBs can be represented below:

$$E(1-e^{-w_{it}}|\varepsilon_{it})=1-\frac{\lambda }{1+\lambda }\frac{\left[\phi (c_{it})+\exp(b_{it}-a_{it})\exp(\sigma_{v}^{2}/2-\sigma_{v}h_{it})\phi (h_{it}-\sigma_{v})\right]}{\exp(b_{it}-a_{it})[\phi (h_{it})+\exp(a_{it}-b_{it})\phi (c_{it})]}$$ (10)

$$E(1-e^{-u_{it}}|\varepsilon_{it})=1-\frac{\lambda }{1+\lambda }\frac{\left[\phi (h_{it})+\exp(a_{it}-b_{it})\exp(\sigma_{v}^{2}/2-\sigma_{v}c_{it})\phi (c_{it}-\sigma_{v})\right]}{\phi (h_{it})+\exp(a_{it}-b_{it})\iota (c_{it})}$$ (11)

Here, the *NTS* denotes the difference between the occupied surplus by IBs and executives, and it can be expressed as follows:

$$NTS=E(1-e^{-u_{it}}|\varepsilon_{it})‐E(1-e^{-w_{it}}|\varepsilon_{it})=E(e^{-w_{it}}‐e^{-u_{it}}|\varepsilon_{it})$$ (12)

Next, the occupied surplus by executives and IBs and *NTS* were quantitatively estimated through the two-tier stochastic frontier and endogenous correction model.

### Selection and definition of variables

*S* denotes the response variable, which is measured as the natural logarithm of the total MC of executives. Referring to previous studies, the following explanatory variables are constructed: (1) capability characteristics of executives, including age (*AGE*), degree of education (*DEGR*), reputation (*TENU*), gender (*GEND*), and financial work experience (*FBAC*); (2) job characteristics of executives, including members of the board (*MTB*), independent directors (*DREC*), employee supervisors (*SSP*), normal supervisors (*NSP*), members of the management team (*MTMT*), chairman of the board (*CTB*), chairman of the board of supervisors (*CSO*), the general manager (*CEO*); (3) enterprise characteristics of IBs, including company scale (*SIZE*), asset-liability ratio (*LEV*), company value (*TOBIN*), profitability (*ROE*), equity balance (*ZDEX*), equity concentration (*HHI3*), and property state (*STAT*). The model systematically controls dummy variables reflecting the regional difference and time effect. The detailed definitions of these variables are listed in Table 1.

**Table 1:** Variable definition.

Variable	Definition
*S*	Ln(the total MC of the executive)
*AGE*	Ln(the age of the executive)
*DEGR*	If the executive has a master degree or doctor degree, the value of *DEGR* is 1; otherwise, 0
*TENU*	Ln(the total number of months of the executive in the current position)
*GEND*	If the executive is female, the value of *GEND* is 1; otherwise, 0
*FBAC*	If the executive has financial work experience, the value of *FBAC* is 1; otherwise, 0
*MTB*	If the executive is a member of the board, the value of *MTB* is 1; otherwise, 0
*DREC*	If the executive is an independent director, the value of *DREC* is 1; otherwise, 0
*SSP*	If the executive is an employee supervisor, the value of SSP is 1; otherwise, 0
*NSP*	If the executive is a normal supervisor, the value of NSP is 1; otherwise, 0
*MTMT*	If the executive is a management team member, the value of *MTMT* is 1; otherwise, 0
*CTB*	If the executive is the chairman of the board, the value of *CTB* is 1; otherwise, 0
*CSO*	If the executive is the chairman of the board of supervisors, the value of *CSO* is 1; otherwise, 0
*CEO*	If the executive is a general manager, the value of *CEO* is 1; otherwise, 0
*SIZE*	Ln(total IB assets)
*LEV*	Ln(asset-liability ratio)
*TOBIN*	Ln(IB market value/ IB book value)
*ROE*	Ln[1+(the average annual balance of net profit/ stockholders’ equity)]
*ZDEX*	Ln(shareholding ratio of the largest shareholder/ shareholding ratio of the second largest shareholder)
*HHI3*	Ln(the Herfindahl index of shares held by the top three shareholders of IBs)
*STAT*	If the IB is state-owned, the value of *STAT* is 1; otherwise, 0

The definition of 14 dummy variables reflecting regional differences and 11 variables reflecting time effects is not presented due to the limited space.

### Sample selection and data sources

This study selected the sample data of IBs in Shanghai and Shenzhen stock markets from 2008 to 2020, and the original data were collected from the Wind database. The shareholding sample of the executives was excluded. Then, Heckman’s two-stage method was employed to overcome the sample selection bias. The missing observations were omitted. Next, all continuous variable sample data were winsorized at the 1% and 99% quantiles. Finally, 6835 samples were retained (see S1 Dataset for more details), which ensured the representativeness of this research.

## Empirical results and analysis

### Variable descriptive statistics

Table 2 presents the descriptive statistics results of the variables used in this study. The difference between the minimum and maximum of the total compensation was obvious, and its standard deviation was 1.581, indicating a significant compensation difference among executives in our sample. Meanwhile, the mean and median of the main variables were close, and the standard deviation was generally small, which indicated that the main variables tend to obey the normal distribution and there was no obvious outlier problem in the sample data.

**Table 2:** Variable descriptive statistics.

Variable	Obs.	Mean	Std. Dev.	Min	Median	Max
*S*	6835	13.14	1.58	9.21	13.55	15.71
*AGE*	6835	3.91	0.15	3.22	3.91	4.41
*AGE2*	6835	15.32	1.17	10.36	15.30	19.42
*DEGR*	6835	0.24	0.43	0.00	0.00	1.00
*TENU*	6835	1.29	0.71	0.00	1.39	2.64
*GEND*	6835	0.13	0.34	0.00	0.00	1.00
*FBAC*	6835	0.81	0.40	0.00	1.00	1.00
*MTB*	6835	0.42	0.49	0.00	0.00	1.00
*DREC*	6835	0.23	0.42	0.00	0.00	1.00
*SSP*	6835	0.12	0.33	0.00	0.00	1.00
*NSP*	6835	0.06	0.23	0.00	0.00	1.00
*MTMT*	6835	0.41	0.49	0.00	0.00	1.00
*CTB*	6835	0.05	0.21	0.00	0.00	1.00
*CSO*	6835	0.04	0.19	0.00	0.00	1.00
*CEO*	6835	0.06	0.23	0.00	0.00	1.00
*SIZE*	6835	24.81	1.45	20.57	24.90	27.49
*LEV*	6835	-0.43	0.29	-1.71	-0.32	-0.13
*TOBIN*	6835	0.37	0.39	-0.62	0.26	2.75
*ROE*	6835	0.07	0.07	-0.25	0.06	0.30
*ZDEX*	6835	0.94	0.92	0.00	0.63	3.97
*HHI3*	6835	-1.40	2.19	-4.55	-2.19	4.23
*STAT*	6835	0.68	0.47	0.00	1.00	1.00

### Regression results of the first stage of Heckman’s two-stage method

Table 3 presents the detailed results of the first stage of Heckman’s two-stage method. Based on the research work performed by Heckman (1979) [40], this study employed the stepwise regression method to eliminate insignificant variables. Next, the setting form of model (4) is retained, and the *IMR* can be calculated based on the regression results of model (4). Finally, the *IMR* was compensated into the two-tier stochastic frontier model.

**Table 3:** The regression results of the first stage of Heckman’s two-stage method.

Variable	Model (1)		Model (2)		Model (3)		Model (4)	
*AGE*	-5.43	(0.46)	-4.82	(0.50)				
*AGE* ^*2*^	0.69	(0.47)	0.61	(0.51)				
*DEGR*	-0.42***	(0.00)	-0.42***	(0.00)	-0.42***	(0.00)	-0.41***	(0.00)
*TENU*	0.39***	(0.00)	0.39***	(0.00)	0.39***	(0.00)	0.40***	(0.00)
*GEND*	-0.29**	(0.01)	-0.29**	(0.01)	-0.29**	(0.01)	-0.26**	(0.02)
*FBAC*	-0.34***	(0.00)	-0.34***	(0.00)	-0.34***	(0.00)	-0.34***	(0.00)
*MTB*	0.34***	(0.00)	0.36***	(0.00)	0.36***	(0.00)	0.28***	(0.00)
*DREC*	-0.78***	(0.00)	-0.77***	(0.00)	-0.76***	(0.00)	-0.78***	(0.00)
*SSP*	0.12	(0.35)	0.15	(0.23)	0.15	(0.23)		
*NSP*	-0.12	(0.57)						
*MTMT*	0.57***	(0.00)	0.59***	(0.00)	0.58***	(0.00)	0.50***	(0.00)
*CTB*	0.36***	(0.00)	0.36***	(0.00)	0.35***	(0.00)	0.33***	(0.01)
*CSO*	0.19	(0.28)	0.22	(0.20)	0.21	(0.21)		
*CEO*	-0.09	(0.46)	-0.11	(0.39)	-0.11	(0.37)		
*SIZE*	-0.10**	(0.03)	-0.11***	(0.01)	-0.12***	(0.00)	-0.07**	(0.03)
*LEV*	-0.08	(0.59)						
*TOBIN*	-0.26*	(0.09)	-0.27*	(0.07)	-0.27*	(0.08)		
*ROE*	1.07	(0.16)	1.14	(0.13)	1.09	(0.14)		
*HHI3*	-0.23***	(0.00)	-0.23***	(0.00)	-0.23***	(0.00)	-0.20***	(0.00)
*STAT*	-0.26**	(0.03)	-0.26**	(0.03)	-0.27**	(0.03)	-0.22*	(0.07)
*_cons*	9.85	(0.48)	8.86	(0.52)	-0.49	(0.63)	-1.55**	(0.04)
*Λ*_*dist*_	Yes		Yes		Yes		Yes	
*Τ*_*time*_	Yes		Yes		Yes		Yes	
*Pseudo-R* ^*2*^	0.35		0.35		0.35		0.35	
*N*	7260		7260		7260		7260	

***, **, and * denote significance levels at 1%, 5%, and 10%, respectively. The *p* values in parentheses are calculated according to the robust standard error.

### Regression results of the two-tier stochastic frontier and endogenous correction model

Table 4 presents the estimation results of the two-tier stochastic frontier and endogenous correction model. All the models in Table 4 systematically control the time and regional effects. Specifically, the variables of executives’ capability characteristics are controlled in model (5). The variables of executives’ capability characteristics are then added to model (6). Furthermore, the variables of IB characteristics are introduced in model (7). The *IMR* is added to model (8) for modifying model (7). The regression results revealed that the likelihood value of model (8) was the largest, validating the superiority of model (8). The likelihood ratio test results of models (5) and (8) also supported the conclusion, which implied the existence of endogenous problems caused by sample selection bias, and the endogenous problem can be eliminated by the two-tier stochastic frontier and endogenous correction model. Next, the results based on model (8) are discussed.

**Table 4:** The regression results of two-tier stochastic frontier and endogenous correction model.

Variable	Model (5)		Model (6)		Model (7)		Model (8)	
*AGE*	80.80***	(0.00)	16.28***	(0.00)	8.92***	(0.00)	9.12***	(0.00)
*AGE* ^2^	-10.59***	(0.00)	-2.04***	(0.00)	-1.13***	(0.00)	-1.16***	(0.00)
*DEGR*	0.26***	(0.00)	0.14***	(0.00)	0.06**	(0.01)	0.07**	(0.01)
*TENU*	0.19***	(0.00)	0.09***	(0.00)	0.07***	(0.00)	0.21***	(0.00)
*GEND*	-0.18***	(0.00)	-0.04*	(0.06)	-0.07***	(0.00)	-0.14***	(0.00)
*MTB*			-0.64***	(0.00)	-0.51***	(0.00)	-0.38***	(0.00)
*DREC*			-1.48***	(0.00)	-1.61***	(0.00)	-1.85***	(0.00)
*NSP*			-2.55***	(0.00)	-2.61***	(0.00)	-2.57***	(0.00)
*MTMT*			0.62***	(0.00)	0.60***	(0.00)	0.74***	(0.00)
*CTB*			1.21***	(0.00)	1.09***	(0.00)	1.16***	(0.00)
*CSO*			0.36***	(0.00)	0.37***	(0.00)	0.38***	(0.00)
*CEO*			0.68***	(0.00)	0.62***	(0.00)	0.60***	(0.00)
*SIZE*					0.20***	(0.00)	0.17***	(0.00)
*LEV*					-0.15***	(0.00)	-0.14***	(0.00)
*TOBIN*					0.17***	(0.00)	0.15***	(0.00)
*ROE*					1.51***	(0.00)	1.27***	(0.00)
*ZDEX*					-0.05***	(0.00)	-0.06***	(0.00)
*STAT*					-0.04*	(0.10)	-0.08***	(0.00)
*IMR*							0.93***	(0.00)
*_cons*	-140.40***	(0.00)	-18.94***	(0.00)	-9.17**	(0.04)	-9.74**	(0.03)
*Λ*_*dist*_	Yes		Yes		Yes		Yes	
*Τ*_*time*_	Yes		Yes		Yes		Yes	
*LL*	-11867.99		-8753.43		-8473.54		-8434.56	
*Chi*_*1*_ *-square*	6866.88		637.75		77.97			
*P*_*1*_ *-value*	0.00		0.00		0.00			
*Chi*_*2*_ *-square*			6229.13		6788.91		6866.88	
*P*_*2*_ *-value*			0.00		0.00		0.00	
*N*	6835		6835		6835		6835	

***, **, and * denote significance levels at 1%, 5%, and 10%, respectively. The *p* values in parentheses are calculated according to the robust standard error. Here, LL denotes the likelihood value. *Chi*_*1*_*-square* denotes the chi-square value obtained from the likelihood ratio test by model (8). *Chi*_*2*_*-square* denotes the chi-square value obtained from the likelihood ratio test by model (5).

The regression results indicated that the executive age exhibited a nonlinear effect on executive MC. With the increase in age, the professional ability of executives strengthened, and the MC increased accordingly. When the age was beyond a threshold, the physical function and intelligence of the executives weakened gradually, which decreased their MC. Meanwhile, education degree and reputation positively affected the MC of executives because a high degree of education or a high reputation can enhance their working ability or leadership. Besides, the ceiling effect restricted the career growth of female executives, which led to a considerably lower MC for female executives.

Moreover, it was found that the MC of chairman of the board, chairman of the board of supervisors, general managers, and senior managers was considerably higher, whereas the MC of common directors, independent directors, and common supervisors was significantly lower. The results revealed that for complex and strenuous jobs, the requirements for competence and dedication were higher, which considerably increased the MC of executives. Also, when the job characteristics of executives tended to enhance the importance of the job, the intervention by executives in the design of MC contracts and performance appraisal was stronger, and the MC of executives increased.

Finally, the results in Table 4 illustrated a positive relationship between the company scale and executive compensation. The management of a large-scale company is complex and requires considerable ability and energy, which results in a higher compensation figure. A negative relationship exists between asset-liability ratio and executive compensation. When IBs encounter high financial risks, the compensation of executives in an IB with a high debt ratio was considerably lower because the cash flow is constrained and the economic performance degrades. Both company value and profitability exhibited a positive effect on the MC, which revealed the sensitivity of executive compensation on performance. Moreover, the equity balance had a positive effect on the MC because a high level of equity balance can improve the corporate governance of IBs and increase the risk of dismissal of executives. Therefore, a higher MC level can significantly offset the possible negative impacts of dismissal risks on executives. Furthermore, because of the stringent regulations to limit the executive compensation of state-owned enterprises, the MC level of executives in state-owned IBs was significantly lower, which resulted in an overall downward trend of executive compensation in state-owned IBs.

### Bargaining mechanism and variance decomposition analysis

Table 5 shows the decomposition results of the compound interference terms of the two-tier stochastic frontier and endogenous correction model. The expectation of surplus occupied by IBs and executives was 0.88 and 0.37, respectively. Thus, the occupied surplus of IBs was 0.51 higher than that of executives on average. This result implies that the bargaining effect reduced the negotiated MC of executives. Meanwhile, the sum of the variance of the occupied surplus accounted for 99.18% of the compound interference term, indicating a significant influence of the bargaining effect on MC decisions. Besides, the proportion of the surplus occupied by IBs was 85.17%, whereas that occupied by executives was 14.83%. Thus, IBs generally had stronger bargaining power than executives, leading to a decrease in the negotiated MC of IB executives.

**Table 5:** Bargaining mechanism and variance decomposition analysis.

Variable meaning	Symbol	Coefficient
Expectation of common stochastic interference term	*σ*_*v*_	0.09
Expectation of occupied surplus by IB	*σ*_*u*_	0.88
Expectation of occupied surplus by executives	*σ*_*w*_	0.37
Sum of variance of compound stochastic interference term	*σ*_*v*_^2^+*σ*_*u*_^2^+*σ*_*w*_^2^	0.92
Contribution of occupied surplus effect	(*σ*_*u*_^2^+*σ*_*w*_^2^)/ (*σ*_*v*_^2^+*σ*_*u*_^2^+*σ*_*w*_^2^)	99.18%
Contribution of the effect of occupied surplus by IBs	(*σ*_*u*_^2^)/ (*σ*_*u*_^2^+*σ*_*w*_^2^)	85.17%
Contribution of the effect of occupied surplus by executives	(*σ*_*w*_^2^)/ (*σ*_*u*_^2^+*σ*_*w*_^2^)	14.83%

### Bargaining effect estimation and analysis

Theoretical model (10) was used to measure the surplus occupied by executives, thus revealing the extent of increased negotiated MC by executives’ bargaining. Also, the surplus occupied by IBs was calculated using theoretical model (11) to illustrate the extent of decreased negotiated MC by IBs’ bargaining. Additionally, theoretical model (12) was used to estimate the *NTS*, which is equal to the difference between the surplus occupied by IBs and executives, to reveal the comprehensive bargaining effect between the IBs and executives on the negotiated MC. Table 6 presents the estimation results of the bargaining effect. On average, the bargaining effect of IBs caused the negotiated MC 46.35% to be lower than the benchmark MC, whereas the bargaining effect of executives caused the negotiated MC to be 26.73% higher than the benchmark MC. That is, because the bargaining power of IBs is stronger than that of executives, the comprehensive bargaining effect decreased the negotiated MC, which caused the negotiated MC to be 19.62% lower than the benchmark.

**Table 6:** Estimation of the occupied surplus effect and descriptive statistics.

Surplus category	Mean	Std. Dev.	Min	Median	Max	T-Test
*IBAN*	46.35%	25.27	20.62%	39.76%	99.43%	48.61***
*SENI*	26.73%	12.73	20.62%	20.63%	97.58%	
*NTS*	19.62%	33.37	-76.95%	19.13%	78.81%	

*** denote a significance level at 1%. *IBAN*, *SENI*, *and NTS* denote the surplus occupied by IBs and executives and the net surplus, respectively. The T-Test is used to test the significance of the difference between *IBAN* and *SENI*.

**Fig 3:**
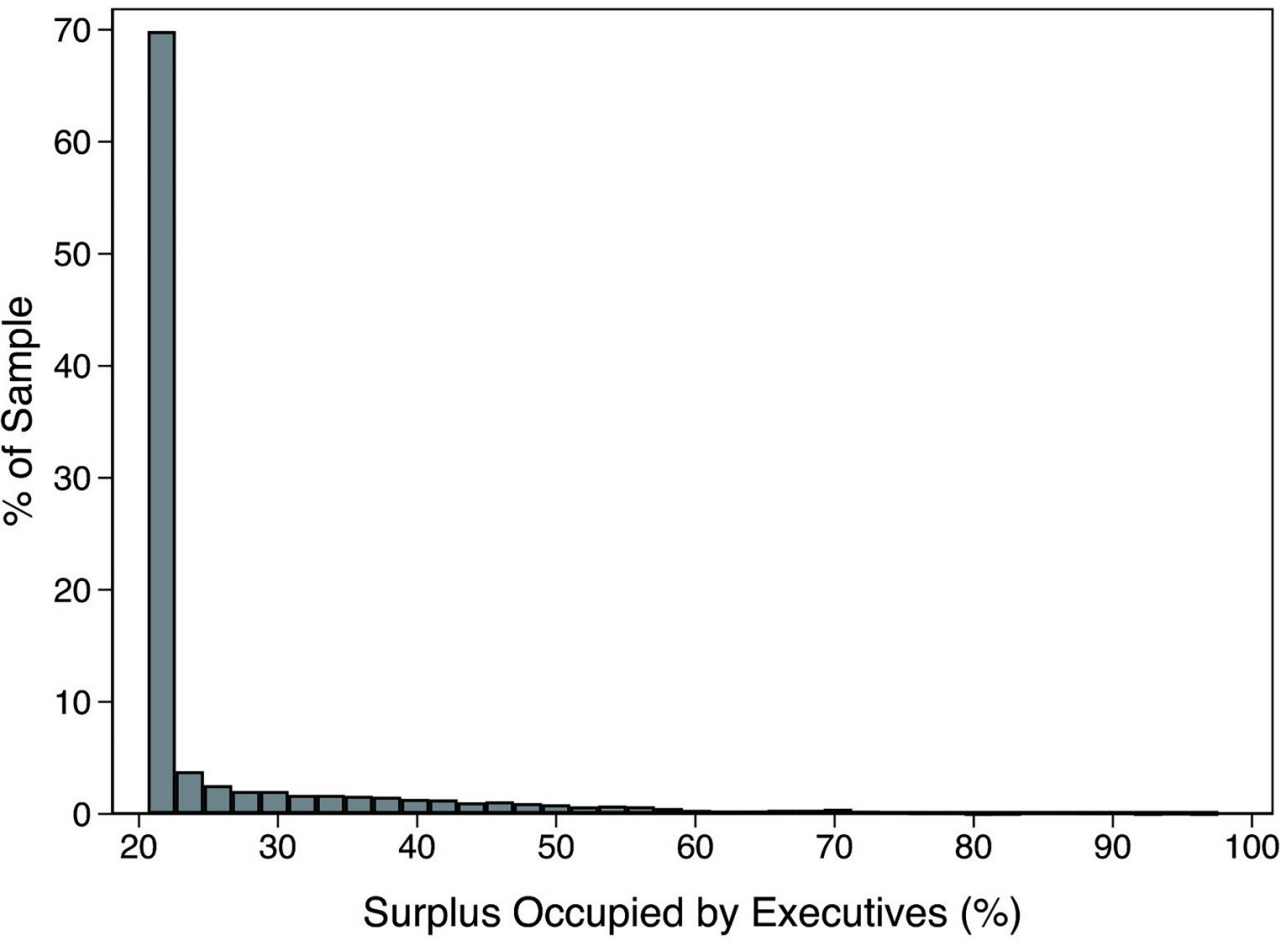
The frequency distribution of the surplus occupied by executives.

**Fig 4:**
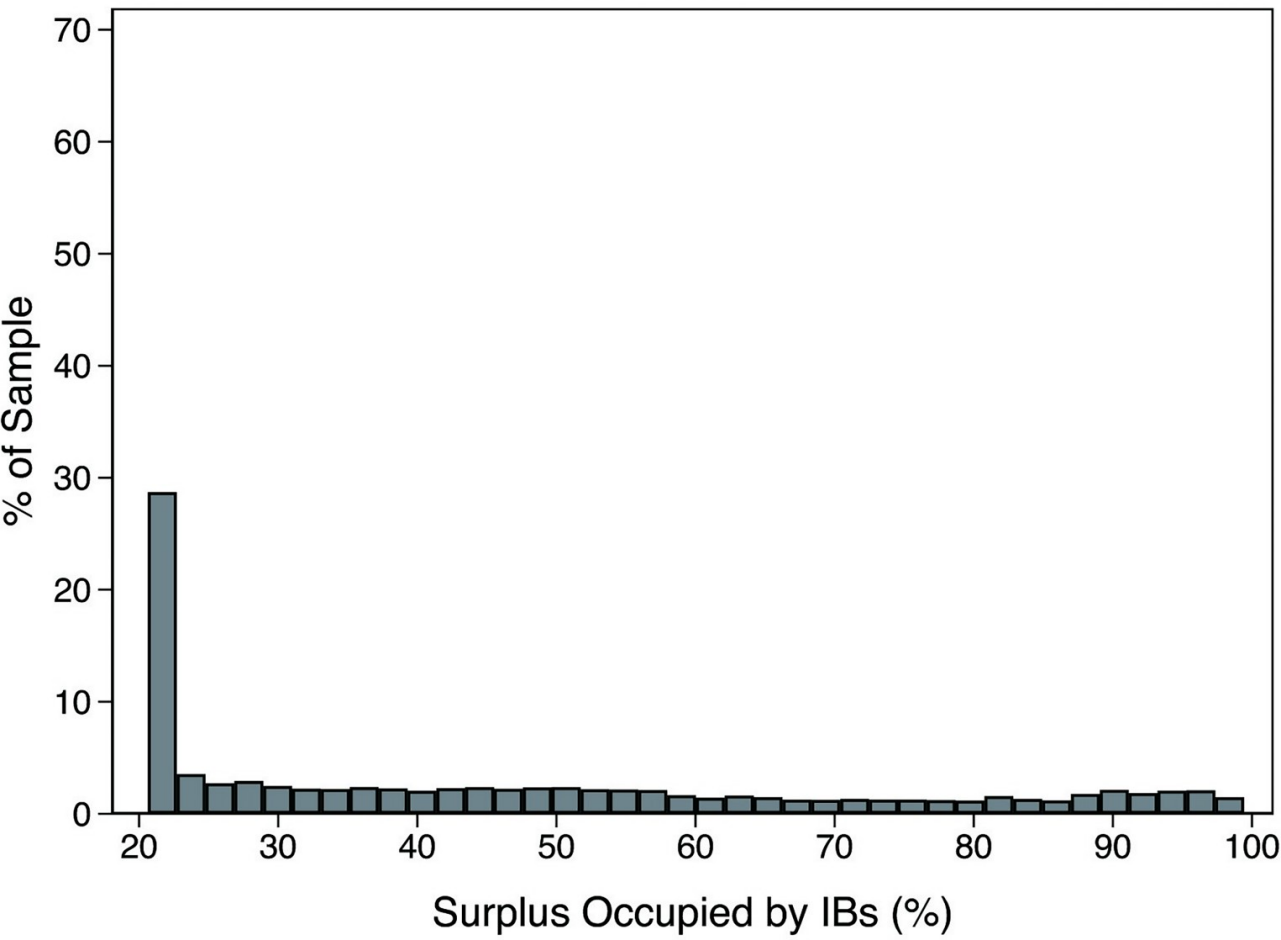
The frequency distribution of the surplus occupied by IBs.

Figs 3 and 4 display the frequency distribution of the surplus occupied by executives and IBs, respectively. The surplus occupied by executives was concentrated at low levels, whereas that occupied by IBs distributed at the high level. These results provide further support for the conclusion that IBs have stronger bargaining power and a higher occupied surplus level than executives. However, either the occupied surplus of IBs or executives presented a tail on the right side, indicating that either would be in an absolute bargaining-dominant state only in a few cases.

**Fig 5:**
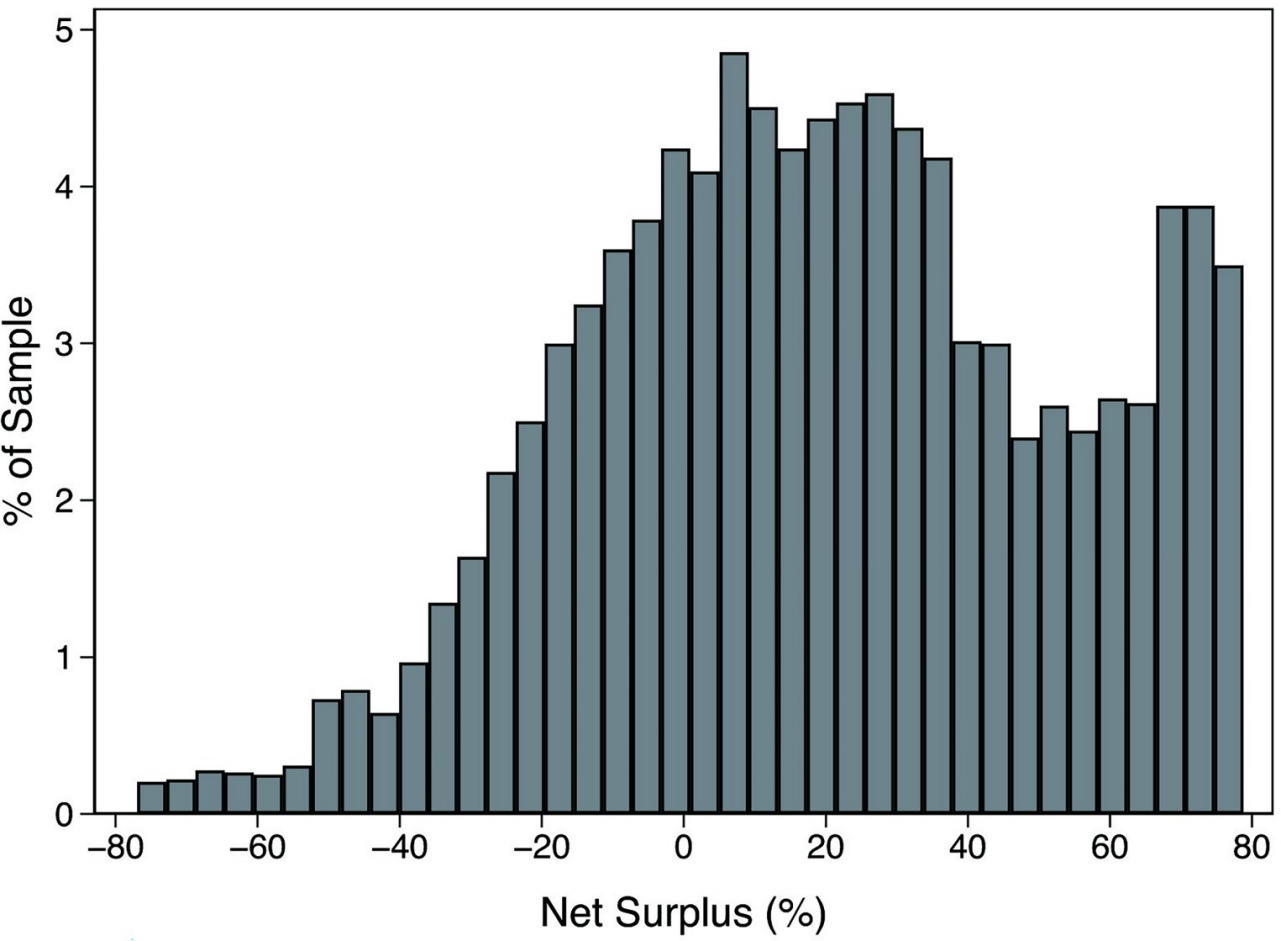
The frequency distribution of the net surplus.

The frequency distribution of the *NTS* presented in Fig 5 demonstrates the distribution characteristics of *NTS*. The result reveals that *NTS* is concentrated more on the right side of the zero axis. Specifically, statistics revealed that the number of samples on the left and right sides accounted for about 29.305% and 70.695%, respectively, i.e., approximately 70.695% of the samples’ negotiated MC was lower, and approximately 29.305% was increased. These findings suggest that the bargaining effects on executive compensation exhibited obvious heterogeneity.

### Further analysis

The frequency distribution of the bargaining effect on the IB executives’ MC decisions illustrated potential heterogeneity. This study selected the characteristics of executives and IBs to analyze the heterogeneity in the bargaining effect.

### Heterogeneity analysis based on the characteristics of executive

The heterogeneity analysis results of the bargaining effect based on executive characteristics are presented in Table 7. The results revealed that: (1) compared with the surplus of young executives, the surplus of senior executives occupied by IBs was considerable, whereas the surplus of IBs occupied by senior executives was limited. Because the physical function and intelligence of the senior executives weakened gradually, their ability to search and exploit the information of MC decisions may be relatively limited. In this case, the power of occupying surplus by senior executives may be weaker, which may decrease the negotiated MC considerably. (2) Compared with low-reputation executives, high-reputation executives could occupy more surplus from IBs, whereas the surplus of executives occupied by IBs was limited. Since executives with considerable experience in their current position are highly regarded, they can easily hide unfavorable information about their working ability and effort; also, they can accumulate significantly more private information about the MC decisions of IBs. Thus, the negotiated MC of highly regarded executives exhibits a limited decrease. (3) The average surplus of highly educated executives occupied by IBs was considerably limited, while the average surplus of IBs occupied by highly educated executives was considerable. Highly educated executives can fully utilize the MC decision-making information, so their average negotiated MC does not decrease considerably. (4) The average surplus of female executives occupied by IBs was more than that of male executives. The ceiling effect restricted the career growth (i.e., a lower position) of female executives, leading to inadequate access to information on MC decisions. Thus, the bargaining power of female executives is limited, and the average negotiated MC of female executive declines considerably. (5) Compared with other bargainers, IBs could occupy more surplus from board members or supervisory board members, whereas these executives occupy less surplus of IBs. Thus, the average surplus of these executives occupied by IBs was high. Since most MC designers are board members or supervisory board members, they know the working abilities and efforts of other members well, which augments the bargaining power of IBs. Thus, the negotiated MC of board members or supervisory board members could decline considerably. (6) Compared with the average surplus of nonmanagement team members, the average surplus of management team members occupied by IBs was considerably lower, and the average surplus of IBs occupied by management team members was higher. Since management team members have complete information about their working ability and efforts while IBs lack information, the bargaining power of IBs weakened. Thus, the average surplus of the management team members occupied by IBs was low, indicating that the decrease in the average negotiated MC by IBs was limited. (7) The average surplus of general managers, chairmen, and chief supervisors occupied by the IB was considerably lower than that of other bargainers, and the average surplus of IBs occupied by these executives was considerably higher. As a result, they obtained a larger *NTS* on average. This finding demonstrates that the comprehensive bargaining effect increases the MC of general managers, chairmen, and chief supervisors. As top executives, major decision-makers and supervisors have the maximum private information on the MC decisions of IBs, which improves their bargaining power and their average negotiated MC.

**Table 7:** Heterogeneity analysis of the bargaining effect based on characteristics of executives.

Variable	Surplus	Attribute	Mean	*K-W*	Variable	Surplus	Attribute	Mean	*K-W*
*AGE*	*IBAN*	Senior	46.28	16.70***	*TENU*	*IBAN*	High	44.99	36.14***
		Young	49.57				Low	49.17	
	*SENI*	Senior	26.29	12.99***		*SENI*	High	26.82	18.43***
		Young	26.22				Low	26.33	
	*NTS*	Senior	19.99	16.21***		*NTS*	High	18.17	35.81***
		Young	23.35				Low	22.85	
*DEGR*	*IBAN*	High	43.27	29.45***	*GEND*	*IBAN*	Female	48.44	12.45**
		Low	47.13				Male	46.24	
	*SENI*	High	28.10	16.62***		*SENI*	Female	25.63	17.68***
		Low	26.78				Male	26.97	
	*NTS*	High	15.17	28.85***		*NTS*	Female	22.81	12.12***
		Low	20.35				Male	19.27	
*MTB*	*IBAN*	Yes	49.68	44.91***	*MTMT*	*IBAN*	Yes	42.19	251.59***
		No	44.78				No	52.34	
	*SENI*	Yes	25.18	9.53***		*SENI*	Yes	27.33	165.80***
		No	26.50				No	24.94	
	*NTS*	Yes	24.50	45.36***		*NTS*	Yes	14.86	248.69***
		No	18.27				No	27.41	
*NSP*	*IBAN*	Yes	88.83	697.99***	*CTB*	*IBAN*	Yes	36.69	132.65***
		No	48.07				No	47.75	
	*SENI*	Yes	19.40	323.92***		*SENI*	Yes	40.78	153.89***
		No	22.85				No	26.02	
	*NTS*	Yes	69.43	698.07***		*NTS*	Yes	-4.09	141.57***
		No	25.22				No	21.73	
*CEO*	*IBAN*	Yes	40.31	53.60***	*CSO*	*IBAN*	Yes	38.93	45.51***
		No	47.07				No	47.01	
	*SENI*	Yes	32.75	48.04***		*SENI*	Yes	31.45	51.12***
		No	26.13				No	26.29	
	*NTS*	Yes	7.55	52.45***		*NTS*	Yes	7.48	44.28***
		No	20.95				No	20.72	
		Low	19.53				Low	18.14	

*** and ** represent significance levels at 1% and 5%. When the categorical variable is continuous, grouping is performed according to the mean of the variable. *IBAN*, *SENI*, *and NTS* denote the surplus of IBs and executives and the net surplus, respectively. *K–W* is the abbreviation of the Kruskal–Wallis test.

### Heterogeneity analysis based on characteristics of IBs

Table 8 presents the heterogeneity analysis results of the bargaining effect based on IBs characteristics. The results reveal that: (1) large-scale IBs occupy more average surplus of executives than small-scale IBs, whereas executives occupy less average surplus of large-scale IBs. Thus, the average *NTS* of executives in large-scale IBs is significantly higher. Executive positions in large-scale IBs are more attractive in human resource markets, which encourages IBs to reduce the maximum expected MC and lower the minimum expected MC of executives. Thus, the surplus of executives occupied by IBs was expanded. As a result, the average negotiated MC of executives in large-scale IBs declines considerably. (2) The average surplus of executives occupied by state-owned IBs was lower than that by private IBs. State-owned IBs exhibited ambiguous property rights, and their governance had several loopholes. This phenomenon negatively affected the enthusiasm and ability of state-owned IBs to bargain. Therefore, the power of occupying surplus by state-owned IBs could weaken, and the negotiated MC of executives in state-owned IBs could decrease marginally. (3) The average surplus of executives occupied by IBs with a high profitability or low financial risk was low, whereas the average surplus of IBs occupied by executives was high. When IBs become profitable or encounter low financial risks, executives attribute these operating performances to their efforts and achieve strong bargaining with IBs on MC decisions; in this case, the bargaining power of IBs is weakened. Thus, the negotiated MC does not reduce considerably when IBs do not exhibit high profitability or low financial risks.

**Table 8:** Heterogeneity analysis of the bargaining effect based on characteristics of IBs.

Variable	Surplus	Attribute	Mean	*K-W*	Variable	Surplus	Attribute	Mean	*K-W*
*SIZE*	*IBAN*	Large	41.64	6.88***	*STAT*	*IBAN*	SOE	39.58	27.80***
		Small	40.38				POE	43.38	
	*SENI*	Large	32.01	21.18***		*SENI*	SOE	33.98	9.22***
		Small	35.01				POE	32.49	
	*NTS*	Large	9.63	16.71***		*NTS*	SOE	5.60	34.59***
		Small	5.38				POE	10.90	
*ROE*	*IBAN*	High	45.84	4.41**	*LEV*	*IBAN*	High	47.06	7.44**
		Low	46.69				Low	45.11	
	*SENI*	High	27.81	6.15***		*SENI*	High	26.53	7.41***
		Low	27.16				Low	26.97	
	*NTS*	High	18.03	4.21**		*NTS*	High	20.53	7.54***
		Low	19.53				Low	18.14	

*** and ** represent significance levels at 1% and 5%. When the categorical variable is continuous, grouping is performed according to the mean of the variable. *IBAN*, *SENI*, and *NTS* denote the surplus occupied by IBs and executives and the net surplus, respectively. *K–W* test is the abbreviation of the Kruskal–Wallis test.

## Conclusion

In this study, a two-tier stochastic frontier and endogenous correction model was developed to test the bargaining effect on MC decisions of executives in IBs. The bargaining effect accounted for 99.18% of stochastic interference information, i.e., the unexplained information affecting the MC of executives. The results revealed that bargaining between executives and IBs considerably affects executive compensation decisions. On average, IBs are highly proficient at bargaining than executives, and the comprehensive bargaining effect reduces the executive compensation, causing the negotiated compensation to be 19.62% lower than the benchmark compensation. Furthermore, the bargaining effect exhibits obvious heterogeneity in executive and IB characteristics. When these characteristics tend to augment the bargaining power of executives, the negotiated MC exhibits a limited decrease; when these characteristics augment the bargaining power of IBs, the negotiated MC markedly decreases.

The study results provide deep insight into factors that determine executive compensation and help compensation designers design appropriate executive compensation packages for IBs. Particularly, this paper has three main implications.

1. Executive compensation contract designers in IBs should comprehensively consider the bargaining effect on the design of executive compensation contracts. In practice, when the complete information condition cannot be satisfied, the bargaining behavior affects the design of the compensation contract. First, to alleviate the drastic deviation from the optimal compensation contract, IBs should be aware that executives with strong bargaining power significantly increase their compensation through their bargaining behaviors. Second, contract designers should avoid excessive bargaining that excessively decreases executive compensation because this may lead to compensation contract design practices significantly lower than the optimal compensation contract. Third, given that this study focuses on Chinese IBs, China’s labor market exhibits features of a buyer’s market, with employer-led labor relations dominant [41]. In this context, empirical evidence reveals that the bargaining effect decreases executive compensation in Chinese IBs. This phenomenon suggests that contract designers should avoid adopting aggressive tactics in bargaining because such tactics could excessively lower executive compensation and cause serious internal principal-agent problems and reduce operating efficiency. Furthermore, IBs should be encouraged to alleviate the information asymmetry problem, especially in relation to compensation design, and enhance the functioning of internal governance mechanisms.
2. To avoid the negative effect of bargaining on the design practice of executive compensation contracts, the influence of heterogeneity generated by the bargaining effect on compensation contracts should be reviewed by IB contract designers. Our empirical results indicated that the impact of bargaining on the compensation contract design practices of IBs is heterogeneous. Thus, contract designers should consider the bargaining effect, which causes the negotiated compensation to be lower than the optimal compensation. This effect is particularly pronounced in specific executive groups, including senior, low-reputation, non-highly educated, female, board, supervisory board, and non-management team members. The interests of these executive groups should be comprehensively considered in the design of compensation contracts. Moreover, the negative effect of bargaining on executive compensation is obvious in IBs with a large scale, privately held, low profitability, and high financial risks. Thus, contract designers should consider the characteristics of IBs when investigating the bargaining effect on executive compensation contract design.
3. IB contract designers should ensure that executive compensation contracts are optimally designed, thus alleviating the complex internal principal-agent problem and improving the governance efficiency of IBs. Our results can provide comprehensive empirical evidence of the determinants of the compensation contract as a valuable reference. That is, when the bargaining effect is to be reviewed by the contract designer in setting executive compensation, the optimal executive compensation contract design should focus on i) the personal characteristics of executives, ii) the job characteristics of executives, iii) characteristics of enterprises, and iv) the local economic development level, social development level, and executive compensation policies.

This study has some limitations. First, due to the lack of detailed information about the executive compensation reported by China-listed IBs, compensation information such as welfare, pension, public accumulation funds, and other deferrals compensation cannot be obtained. Since such unavailable compensation components have a small weight, the total MC provides an appropriate picture of executive compensation in IBs. Future studies should collect comprehensive executive compensation data to ensure a scientific measurement of executive pay. Second, considering the difficulty in determining equity-based compensation, the sample of equity-holding executives was excluded. Although our model adopted the Heckman two-stage correction method to address the problem of sample selection bias, future studies should consider these parameters and investigate innovative measurement methods of equity-based compensation to overcome the measurement bias of executive compensation. Third, when evaluating the bargaining effect in compensation decisions, this study did not consider the union’s moderating effects because the role of Chinese unions is highly restricted and their influence in compensation setting is not as effective as expected [41,42]. Incorporating the union’s moderating effect into the analysis of the bargaining effect on executive compensation and exploring the role of the union in the bargaining of executive compensation decisions should be considered in the future. Finally, our institutional context is constrained to China. The Chinese government sets a radical salary regulation policy for high-income executives in the financial industry because equity is an important concern of government regulators. Moreover, the supply of financial management talents exceeds the demand in the large Chinese labor market. These factors affect the bargaining power of IB and executives. Therefore, the study results are applicable to the IBs of China or that of countries with a similar institutional background and labor market as China. In the future, the differences among countries and industries should be considered when investigating the bargaining effect on compensation decisions in other settings.

## Supporting information

S1 Dataset (CSV)
